# Identification of new inhibitors of soluble guanylyl cyclase activity

**DOI:** 10.1186/2050-6511-16-S1-A95

**Published:** 2015-09-02

**Authors:** Jagamya Vijayraghavan, Focco van den Akker

**Affiliations:** 1 Department of Biochemistry, Case Western Reserve University, Cleveland, OH 44106, USA

## Objective

Soluble guanylyl cyclase, (sGC) is an enzyme that can be activated endogenously by nitric oxide (NO). As its particulate counterparts, the role of the cytoplasmic sGC inside the cell is to convert GTP to the second messenger, cGMP. NO is produced by nitric oxide synthase during the conversion of *l-*arginine to *l-*citrulline. NO binds to the ferrous heme of sGC and activates the enzyme, augmenting the production of cGMP which leads to the activation of cGMP-dependent targets. The activation of various proteins through elevated cGMP production by sGC produces physiological functions like vasodilation, inhibition of platelet aggregation, photosensitivity, and cell growth. However, studies have indicated that during pathophysiological conditions like sepsis and cancer, the inhibition of sGC activity could potentially be beneficial in achieving a desired cellular and pharmaceutical response. In addition, a common inhibitor of sGC, ODQ, only inhibits NO-stimulated sGC, not basal, and ODQ also does not work well under high NO concentrations thus furthering the need to develop novel sGC inhibitors.

## Methods

Using the recent structures of the catalytic domain of sGC and the University of Cincinnati compound library, our lab has discovered 3 new inhibitors of sGC using *in silico* high throughput screening followed by cGMP activity assay testing; sGC was stimulated using nitric oxide in this assay. In addition to inhibiting NO-stimulated sGC, two of the inhibitors also inhibited BAY58-2667-stimulated sGC activity. The 3 inhibitors also showed inhibition of purified heterodimeric catalytic domain of sGC strengthening the hypothesis that they act on the catalytic domain of sGC. We also used various biophysical and biochemical assays to check the behaviour of these compounds in solution and inhibition of unrelated protein activities.

## Results

Our results show that our inhibitors inhibit sGC with affinities ranging from 5.8-45μM. We also find that our inhibitors do not aggregate in solution and seem specific to sGC as these inhibitors do not inhibit an unrelated enzyme activity (i.e. β-galactosidase).

## Conclusions

Our observations suggest that our newly discovered inhibitors of sGC could serve as lead molecules for iterative drug design to develop effective inhibitors of sGC. Furthermore, we want to solve the structure of the catalytic domain bound to the inhibitor to determine the interaction between the protein and the inhibitor.

